# Carboxamido steroids inhibit the opening properties of transient receptor potential ion channels by lipid raft modulation[S]

**DOI:** 10.1194/jlr.M084723

**Published:** 2018-08-09

**Authors:** Éva Sághy, Maja Payrits, Tünde Bíró-Sütő, Rita Skoda-Földes, Eszter Szánti-Pintér, János Erostyák, Géza Makkai, György Sétáló, László Kollár, Tamás Kőszegi, Rita Csepregi, János Szolcsányi, Zsuzsanna Helyes, Éva Szőke

**Affiliations:** Department of Pharmacology and Pharmacotherapy,* University of Pécs, Hungary; Department of Medical Biology,§§ University of Pécs, Hungary; Department of Laboratory Medicine,††† University of Pécs, Hungary; Medical School, János Szentágothai Research Center and Centre for Neuroscience,† University of Pécs, Hungary; Department of Experimental Physics,†† University of Pécs, Hungary; Department of Inorganic Chemistry and MTA-PTE Research Group for Selective Chemical Syntheses,*** University of Pécs, Hungary; Department of Pharmacology and Pharmacotherapy, ^§^ Semmelweis University, Budapest, Hungary; Department of Organic Chemistry, Institute of Chemistry,** University of Pannonia, Veszprém, Hungary; National Brain Research Program-2 Chronic Pain Research Group,§§§ Pécs, Hungary

**Keywords:** steroid, lipid rafts, Transient Receptor Potential channel, sensory neuron, nerve terminal, methyl β-cyclodextrin

## Abstract

Transient Receptor Potential (TRP) cation channels, like the TRP Vanilloid 1 (TRPV1) and TRP Ankyrin 1 (TRPA1), are expressed on primary sensory neurons. These thermosensor channels play a role in pain processing. We have provided evidence previously that lipid raft disruption influenced the TRP channel activation, and a carboxamido-steroid compound (C1) inhibited TRPV1 activation. Therefore, our aim was to investigate whether this compound exerts its effect through lipid raft disruption and the steroid backbone (C3) or whether altered position of the carboxamido group (C2) influences the inhibitory action by measuring Ca^2+^ transients on isolated neurons and calcium-uptake on receptor-expressing CHO cells. Membrane cholesterol content was measured by filipin staining and membrane polarization by fluorescence spectroscopy. Both the percentage of responsive cells and the magnitude of the intracellular Ca^2+^ enhancement evoked by the TRPV1 agonist capsaicin were significantly inhibited after C1 and C2 incubation, but not after C3 administration. C1 was able to reduce other TRP channel activation as well. The compounds induced cholesterol depletion in CHO cells, but only C1 induced changes in membrane polarization. The inhibitory action of the compounds on TRP channel activation develops by lipid raft disruption, and the presence and the position of the carboxamido group is essential.

Members of the Transient Receptor Potential (TRP) family are nonselective cation channels that are multisteric receptors activated by the binding of a variety of exogenous ligands and endogenous mediators, as well as temperature changes. Several TRP receptors are located on the sensory neurons and they are key molecules for sensing pain (1–3).

TRP Vanilloid 1 (TRPV1) capsaicin receptor is one of the most important plasma membrane proteins expressed on a large population of polymodal nociceptors and mediates painful signals (4–8). It is gated by noxious heat (>43°C), protons (pH < 6.0), arachidonic acid, or other FA metabolites produced in response to inflammation or tissue injury, as well as by chemical irritants like capsaicin or resiniferatoxin (9–13). Another similar channel, the TRP Ankyrin repeat domain 1 (TRPA1) receptor is mainly colocalized with TRPV1 in the sensory neuronal membrane. Mediators of oxidative stress, inflammation, and pain, e.g., methylglyoxal, formaldehyde, hydrogen peroxide, cold, and mechanical stimuli activate this receptor, besides exogenous compounds, such as cinnamaldehyde, allyl-isothiocyanate (AITC in mustard oil), 4-hydroxynonenal, and allicin (14–23).

TRP Melastatin 3 (TRPM3) is mainly stimulated by the cholesterol derivative neurosteroid pregnenolone sulfate (PS) being the precursor of a wide range of steroid hormones, as well as dihydro-d-erythro-sphingosine, *N*,*N*-dimethyl-d-erythro-sphingosine, and epipregnanolone sulfate (24–28). The presence and stereochemical orientation of the sulfate group of these agonists greatly determines the TRPM3 activation potential and mechanisms. Specific steroid binding site(s) and unique permeation pathway(s) have been suggested for the lipid compounds on TRPM3 (29–32) that contribute to thermonociception and inflammatory heat hyperalgesia (28).

The fourth thermosensitive ion channel of this family is TRP Melastatin 8 (TRPM8), gated by the supercooling agent icilin, menthol, and temperatures below 26°C (33–38).

These TRP channels located not only on the cell bodies, but both central and peripheral terminals of primary sensory neurons, are important regulators of pain and inflammatory processes (39, 40). Their activation on the peripheral endings induce neurogenic inflammation in the innervated area due to the release of vasoactive and proinflammatory neuropeptides, such as calcitonin gene-related peptide and Substance P (41–43). Therefore, these receptors, with specific emphasis on TRPV1, have been in the focus of analgesic and antiinflammatory drug development in the last 2 decades, most importantly for the treatment of chronic neuropathic pain and inflammatory diseases involving substantial neurogenic inflammatory components (arthritis, psoriasis, inflammatory bowel diseases, and chronic obstructive pulmonary diseases) (44–46). These are still considered to be “unmet medical need” conditions because the presently available drugs do not provide satisfactory relief in most cases or they induce severe side effects (47). There is a strong proof of concept that the inhibition of primary sensory neurons is a very effective analgesic and antiinflammatory mechanism, but antagonists of these TRP channels, most importantly of TRPV1, have still not been registered as new drugs due to their hyperthermic side effects leading to their failure in phase II and III clinal trials (48).

Therefore, finding new, alternative mechanisms to selectively inhibit nociceptor function via blocking TRP channels, if possible even simultaneously, might be a potential way for the development of novel analgesic and antiinflammatory drugs. These ion channels, similar to the nicotinic acetylcholine receptor, are surrounded by lipid rafts in the sensory neuronal membrane (49–56). Lipid rafts are cholesterol, SM, and ganglioside-rich microdomains, and elucidating their function has been an emerging topic recently. Controversial data have been described about the role of lipid rafts in the function of TRP channels. Cholesterol depletion with methyl β-cyclodextrin (MCD) incubation caused impaired signaling processes (54–56), diminished the amplitude of capsaicin-induced currents in primary sensory neurons, but had no effect on heat-induced activation in TRPV1-transfected *Xenopus laevis* oocytes (57, 58) or agonist binding to TRPV1 in rat C6 glioma cells (59). MCD treatment influenced the activation potential of TRPM8 in sensory neurons (60). We recently discovered that lipid raft disruption by pharmacological depletion of various constituents, such as SM, cholesterol, or gangliosides, reduced TRPV1, TRPA1, and TRPM8 activation on sensory neurons and a TRPV1-transfected cell line (61, 62).

Several endogenous steroids have been described to inhibit TRPV1. Dehydroepiandrosterone (DHEA) decreases capsaicin-evoked currents in primary sensory neurons (63), but it is not clear whether steroids bind directly to the capsaicin-binding domain or allosterically modulate TRPV1 activation. We published that our synthetic steroid compound (C1) decreased the capsaicin-induced activation of TRPV1 (64), but the mechanism of action and other related TRP channels were not investigated.

Therefore, in the present study, we examined the effect of this C1 molecule on TRPV1, TRPA1, TRPM3, and TRPM8 ion channel activation, in comparison with other structurally related compounds (C2 and C3), specifically addressing potential lipid raft modifying abilities on primary sensory neurons and receptor-expressing cell lines. C1 and C2 have an *N*-(prop-2-ynyl)-carboxamido group; the main difference in the structure of these compounds lies in the unnatural *cis* junction of rings C and D in C1. Compound C3, a 16-keto-18-nor-13α-steroid, was the starting material during the synthesis of C1 (without the *N*-(prop-2-ynyl)-carboxamido group) (**Fig. 1**).

**Fig. 1.**
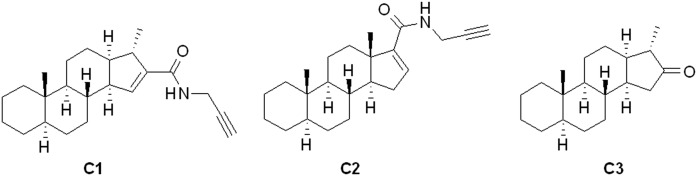
Structure of our steroid compounds. C1 and C2 have a *N*-(prop-2-ynyl)-carboxamido group; the main difference in the structure of these compounds lies in the unnatural *cis* junction of rings C and D in C1. Compound C3, a 16-keto-18-nor-13α-steroid, was the starting material during the synthesis of C1.

With these experiments, our aim was to determine whether and how our novel synthetic steroids having different steroidal skeleton shapes and carboxamido group positions influences the activation of the TRP channels with specific emphasis on membrane lipid rafts. Our results can provide important outcomes from both the lipid raft investigating experimental tool and potential drug developmental points of view.

## MATERIALS AND METHODS

### Primary cultures of trigeminal ganglion neurons

Trigeminal ganglion (TG) cultures were prepared from neonatal 1–3 day old NMRI mice. Ganglia were excised in ice-cold PBS and incubated for 20 min at 37°C in PBS containing collagenase Type XI (1 mg/ml), and then in PBS with DNase I (1,000 units/ml) for 8 min. After washing with PBS, mechanical dissociation was performed. Cells were plated on poly-d-lysine-coated glass coverslips in a medium containing DMEM-low glucose, 5% horse serum, 5% newborn calf serum, 5% FBS, 0.1% penicillin-streptomycin, and 200 ng/ml nerve growth factor (NGF). Cells were maintained at 37°C in a humidified atmosphere with 5% CO_2_ (65).

### Ratiometric technique of intracellular free calcium concentration measurement with the fluorescent indicator fura-2 AM

Measurements were performed on 1–2 day old cell cultures. Cells were incubated for 30 min at 37°C with 1 μM fluorescent Ca^2+^ indicator dye, fura-2-AM in a solution containing (in mM): NaCl, 122; KCl, 3.3; CaCl_2_, 1.3; MgCl_2_, 0.4; KH_2_PO_4_ 1.2; HEPES, 25; and glucose, 10; (pH 7.3). After staining, cells were washed for 5 min with extracellular solution (ECS) containing (in mM): NaCl, 150; KCl, 2.5; CaCl_2_x2H_2_O, 1; MgCl_2_ x 6H_2_O, 2; HEPES, 10; and glucose, 10; (pH 7.3). The rapid changing of solutions from a triplet outlet tube was controlled with a fast step perfusion system (catalog no. VC-77SP, Warner Instrument Corporation, Harvard Apparatus GmbH, Germany). Calcium transients were examined with microfluorimetry as described elsewhere (65). Fluorescent imaging was performed with an Olympus LUMPLAN FI/×20 0.5 W water immersion objective and a digital camera (CCD, SensiCam PCO, Germany). The fluorescence of maximally 12 dye-loaded cells per plate was monitored. Monochromator (Polychrome II., Till Photonics, Germany) generated 340 and 380 nm light (for 50–200 ms each) was used for alternate illumination of cells controlled by Axon Imaging Workbench 2.1 (AIW, Axon Instruments, CA) software. Emitted light (wavelength > 510 nm) was recorded. *R* = F340/F380 was monitored (rate 1 Hz) continuously for up to 2 min. R values generated by AIW 2.1 software were subsequently processed by the Origin software version 7.0 (Originlab Corp.. Northampton, MA). Ratiometric response peak magnitude was measured.

Neurons were incubated with 100 µM C1, C2, and C3 for 45 min, respectively, at 37°C in a humidified atmosphere with 5% CO_2_, or with the solvent of these compounds as control. Additional control experiments with the C1 compound were performed to evaluate the actions on TRPV1 with 7.5% serum (2.5% horse serum, 2.5% newborn calf serum, and 2.5% FBS) during the incubations. Capsaicin (330 nM), AITC (100 µM), icilin (1 µM), and PS (50 µM) were administered for TRPV1, TRPA1, TRPM8, and TRPM3 activation, respectively.

### Radioactive calcium-45 uptake experiments in CHO cells expressing cloned human TRPV1 and TRPA1 receptor

CHO cells stably expressing the TRP receptors were plated into medium onto Microwell Minitrays from Sigma Inc. in 15 μl of cell culture, similarly as described earlier for HT1080 cells (66). Cell lines were prepared in our laboratory, and passed six to seven times. The following day, the cells were washed five times with calcium-free Hank’s solution (pH 7.4), then incubated in 15 μl of the same buffer containing the desired amount of C1, C2, or C3 (10, 50, or 100 µM) at 37°C. After washing with Hank’s solution, the cells were incubated in 10 μl of the same buffer containing 100 nM TRPV1 agonist capsaicin or 100 μM TRPA1 activator AITC and 200 μCi/ml ^45^Ca isotope (1.3 Ci/mmol; Amersham) for 2 min at room temperature. After washing five times with ECS, the residual buffer was evaporated. Then, the retained isotope was collected in 15 μl of 0.1% SDS, and the radioactivity was measured in 2 ml of scintillation liquid in a Packard Tri-Carb 2800 TR scintillation counter.

### Synthesis of steroid compounds

Steroids (Fig. 1) were synthesized by methods described previously in detail (64, 67, 68).

**Fig. 2.**
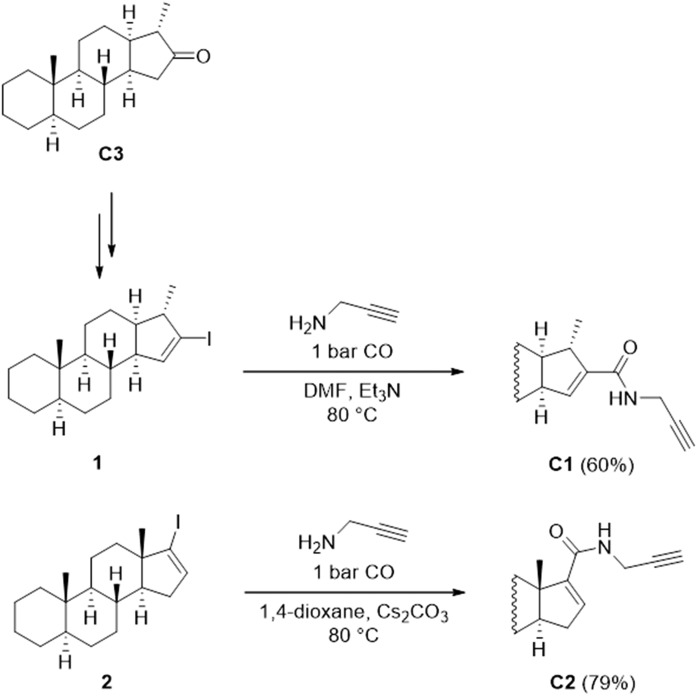
Synthesis of C1 and C2. The iodoalkene mixture was converted to *N*-(prop-2-ynyl)-carboxamides via a palladium-catalyzed aminocarbonylation reaction.

In brief, the C3 was obtained via an unusual Wagner-Meerwein rearrangement of 16α,17α-epoxy-5α-androstane in the presence of imidazolium-based ionic liquids (67). The derivatization of the unnatural C3 was performed by Barton’s methodology, leading to an iodoalkene mixture [16-iodo-16-ene and 16-iodo-15-ene (**1**) derivatives] (64). The iodoalkene mixture was converted to *N*-(prop-2-ynyl)-carboxamides via a palladium-catalyzed aminocarbonylation reaction. The two products were separated from each other by column chromatography; therefore, C1 was obtained in pure form (Fig. 2). Aminocarbonylation reaction of 17-iodo-5α-androst-16-ene (**2**) in the presence of prop-2-yn-1-amine led to steroidal carboxamide C2 of the natural androstane series (68).

#### General procedure for the carbonylation of unnatural steroidal iodoalkenes*.*

A mixture of 16-iodo-15-ene (**1**) and the isomeric 16-iodo-16-ene (in a molar ratio of 55/45), Pd(OAc)_2_, and PPh_3_ were placed under carbon monoxide in a Schlenk tube equipped with a magnetic stirrer, a septum inlet, and a reflux condenser with a balloon on the top. DMF, prop-2-yn-1-amine, and Et_3_N were added through the septum inlet. The product was purified by column chromatography to produce the 16-carboxamido-15-ene steroid C1 in 60% yield [based on the amount of the 16-iodo-15-ene derivative (**1**) in the starting material].

#### General procedure for the carbonylation of steroidal iodoalkene*.*

In a typical procedure, 17-iodo-5α-androst-16-ene (**2**), Cs_2_CO_3_, Pd(OAc)_2_, and PPh_3_ were placed under carbon monoxide in a Schlenk tube equipped with a magnetic stirrer, a septum inlet, and a reflux condenser with a balloon on the top. The 1,4-dioxane and prop-2-yn-1-amine were added through the septum inlet. The reaction was monitored by TLC. Cs_2_CO_3_ was filtered. The product C2 was isolated by column chromatography in 79% yield.

### Filipin fluorescence staining of free cholesterol in CHO cells

Cholesterol content of the CHO cells was analyzed with a cholesterol-binding compound filipin with the same method as described on machrophages (69). For cholesterol depletion, cells that were treated with 10 mM MCD for 45 min at 37°C served as a positive control. Other cells were incubated with 100 µM C1, C2, and C3 for 45 min at 37°C or with the solvent of the compounds. After washing with PBS, cells were fixed with 4% paraformaldehyde for 1 h at room temperature. Cells were washed with PBS and quenched with 1.5 mg/ml glycine in PBS for 10 min at room temperature. Filipin staining (0.05 mg/ml filipin in PBS/10% FBS) was performed for 2 h at room temperature. Cells were then washed three times with PBS, and they were analyzed by fluorescence microscopy. Micrographs have been generated with an Olympus Fluoview-1000 system on an Olympus IX81 microscope stage equipped with an Olympus DP70 digital camera and through an Olympus UPlan FL N, Phase2 objective (40×/0.75). Image size was set at 4080 × 3072 and ISO at 200. Quantification of filipin staining was performed with ImageJ software. Fluorescence intensity of 120 cells was determined in at least three different slides.

### Fluorescence spectroscopy

Detection of 6-dodecanoyl-*N*,*N*-dimethyl-2-naphthylamine’s (laurdan’s) fluorescence emission is an efficient tool to study membrane structures both in model membranes and living cells (70, 71). It was successfully used to distinguish between liquid-ordered and liquid-disordered membrane phases. The spectral position and shape of fluorescence emission and excitation spectra of laurdan depend both on the speed of its dipolar relaxation and the polarity of its microenvironment. TGs are isolated from 12 neonatal (postnatal day 1–3) NMRI mice. TG cultures were incubated with 100 µM C1, C2, and C3 for 45 min at 37°C before laurdan administration (2 µM for 40 min at 37°C). After washing with PBS, cells were scraped from the plates and homogenized. To record fluorescence excitation and emission spectra, a HORIBA Jobin-Yvon Nanolog FL3-2iHR spectrofluorometer equipped with a 450-W xenon lamp was utilized. During the measurements, samples were kept at a constant 20°C using a Thermo Scientific circulating bath AC200-A25 with a 4 mm path length quartz cuvette (Hellma 104F-QS).

#### Excitation-emission matrices.

To clearly visualize spectral changes, excitation and excitation-emission matrices (EEMs) were measured for all samples, which consists of a series of emission spectra measured at different excitation wavelengths. This matrix obtained this way has one axis for the emission wavelengths, whereas the other includes the excitation wavelengths. At the intersection points, fluorescence intensity can be read as the value of the third axis.

#### Anisotropy.

To study the molecular orientation and mobility, steady-state emission anisotropy was measured in “L-format” arrangements for all the samples. The excitation was vertically polarized, while anisotropy was calculated from consecutively measured vertical and horizontal polarized intensities. Anisotropy <*r*> is defined as:

$$\langle r\rangle =\frac{I_{\mathrm{VV}}–G\times I_{\mathrm{VH}}}{I_{\mathrm{VV}}+2\times G\times I_{\mathrm{VH}}},$$

where *G* is the spectrofluorometer’s sensitivity factor given by:

$$G=\frac{I_{\mathrm{HV}}}{I_{\mathrm{HH}}}.$$

*G* value was automatically recalculated at each points of the anisotropy measurement.

### Viability assay

CHO cells in 96-well plates were washed three times with PBS using a Biotek ELx50 Elisa washer programmed to gentle filling and aspiration. Cells were treated with 100 μM C1, C2, and C3 in ECS for 45 min at 37°C and then washed three times with PBS. The drained wells were incubated with 250 μl of 5% perchloric acid (PCA) for 15 min at room temperature. ATP was measured by the luciferin–luciferase technique (72) adapted to microplates (73). Briefly, PCA extracts were neutralized by 9.13% KOH in a separate standard microplate. ATP was measured using a white 96-well optical plate (20 μl neutralized sample/200 μl ATP reagent). Standards in the range of 120 nM to 1 μM were prepared and treated the same way as the samples. Luminescence of the standards/samples was measured by a multimode plate reader (EnSpire, PerkinElmer) with 5 s integration time. The number of attached cells in the wells were assessed by 4′,6-diamidino-2-phenylindole dihydrochloride (DAPI) nucleic acid staining [100 μl/well of 10 μg/ml DAPI solution in McIlvain’s buffer (citric acid/Na_2_HPO_4_, pH 7.0)]. Fluorescence of DAPI was read in the multimode plate reader at 355 nm excitation and 460 nm emission wavelength, with area scan mode (cylindrical scan, 25 measuring points/well). Total intracellular protein of the acid fixed cells in the wells was measured after solubilization by 250 μl of 1 mol/l NaOH for 15 min at room temperature. Extracted proteins were derivatized by 4-phenylspiro-[furan-2(3H),1-phthalan]-3,3′-dione (fluorescamine) in a 0.2 M borate/NaOH buffer containing 0.1% Triton X-100 at pH 9.2 (Spectrophotometry and Spectrofluorimetry). Fluorescence was quantified using the multimode plate reader at 385 nm excitation and 490 nm emission wavelengths, and dilutions of BSA served as standards.

Data obtained for the treated cells were referred to the untreated controls and were expressed in percentage. To obtain further information on the viability of the cells, ATP/DAPI and ATP/protein ratios were also calculated and also expressed in percentage (73).

### Drugs and chemicals

AITC (Sigma, St. Louis, MO) was dissolved in DMSO (Sigma) to obtain 10 mM stock solution. Further dilutions were made with ECS solution to reach final concentrations of 100 μM. Capsaicin (Sigma) was dissolved in DMSO to obtain a 10 mM stock solution. Further dilutions were made with ECS or Hank’s solution to reach final concentrations of 330 or 100 nM, respectively. Icilin and PS were purchased from Sigma and dissolved in ECS to reach final concentrations of 1 and 50 μM, respectively. MCD was purchased from Sigma, dissolved in ECS solution. Filipin III from was obtained from Sigma and dissolved in DMSO to reach 1 mg/ml stock solution. Laurdan was purchased *Streptomyces filipinensis* from Sigma and dissolved in DMSO to obtain a 10 mM stock solution. Penicillin-streptomycin was purchased from Gibco (Grand Island, NY). DMEM-low glucose, collagenase type XI, DNase I, horse serum, newborn calf serum, FBS, poly-d-lysine, glycine, and NGF were purchased from Sigma. C1, C2, and C3 were dissolved in DMSO to obtain 10 mM stock solution. Further dilutions were made with ECS and Hank’s solution. DAPI, fluorescamine, and the ATP CLS II bioluminescent reagent kit were also purchased from Sigma.

### Statistical analysis

Data reported in this paper are the means ± SEM or the means ± SD of at least three independent experiments. Statistical analysis was performed by Kruskal-Wallis test with Dunn’s post hoc test, Student’s *t*-test or one-way ANOVA with Bonferroni’s post hoc test; in all cases, *P* < 0.05 was considered statistically significant.

### Ethics

Mice were kept in the Laboratory Animal House of the Department of Pharmacology and Pharmacotherapy, University of Pécs at 24–25°C and provided standard mouse chow and water ad libitum. All efforts were made to minimize animal suffering and to reduce the number of animals used. All experimental procedures were carried out according to the European legislation (Directive 2010/63/EU) and Hungarian Government regulation (40/2013., II. 14.). The studies were approved by the Ethics Committee on Animal Research of Pécs University according to the Ethical Codex of Animal Experiments, and license was given (license BA02/2000-5/2011).

## RESULTS

### Effect of steroid compounds C1, C2, and C3 on capsaicin-evoked ^45^Ca uptake on CHO cells expressing the cloned TRPV1 receptor

**Fig. 3.**
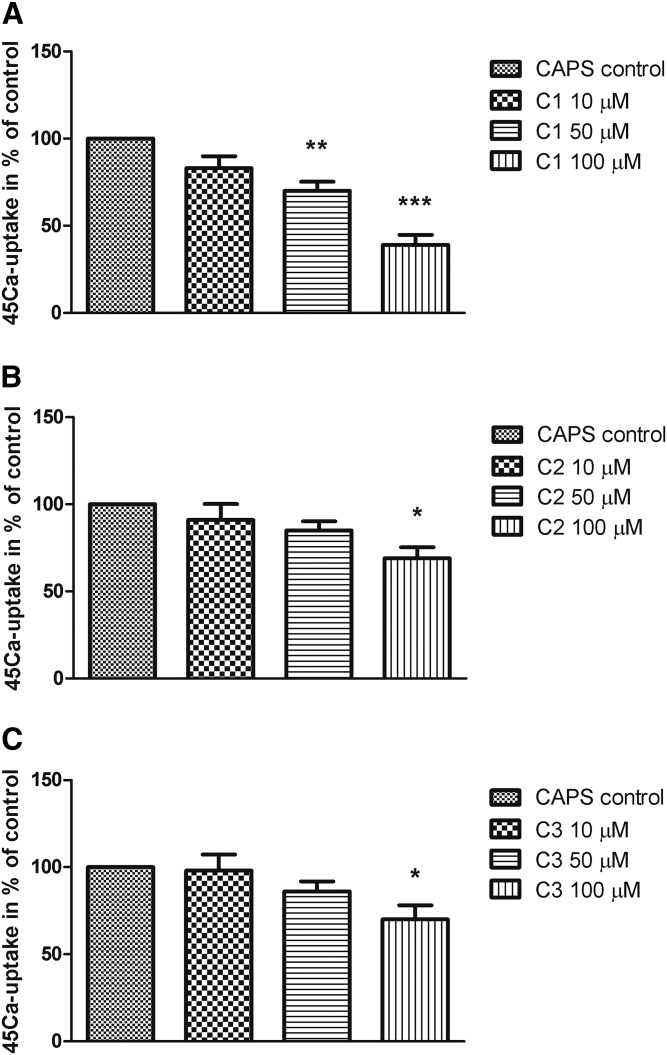
Effect of steroid compounds C1 (A), C2 (B), and C3 (C) (10, 50, and 100 µM, respectively) on TRPV1-expressing CHO cell line in radioactive ^45^Ca-uptake experiments after capsaicin (CAPS, 100 nM) administration. ^45^Ca-accumulations are presented in percent of control (vehicle-treated). Each column represents the mean ± SD of values of three experiments. * *P* < 0.05; ** *P* < 0.01; *** *P* < 0.001 (C1-, C2-, or C3-treated vs. control, one-way ANOVA, Bonferroni’s post hoc test).

In the presence of C1, the intracellular ^45^Ca uptake induced by 100 nM capsaicin was significantly diminished in a concentration-dependent manner. **Figure 3** shows the percent values of ^45^Ca uptake relative to the vehicle control (100%). C1 in 50 and 100 µM concentrations decreased ^45^Ca uptake to 70 ± 9% and 39 ± 10%, respectively. Decrements in capsaicin-induced Ca^2+^ influx were nonsignificant up to treatment with 50 µM C2 and C3, but after 100 µM concentrations, capsaicin responses diminished to 69 ± 11% or 70 ± 14%, respectively.

### Effect of steroid compounds C1, C2, and C3 on TRPV1 receptor activation-mediated Ca^2+^ influx in cultured TG neurons

**Fig. 4.**
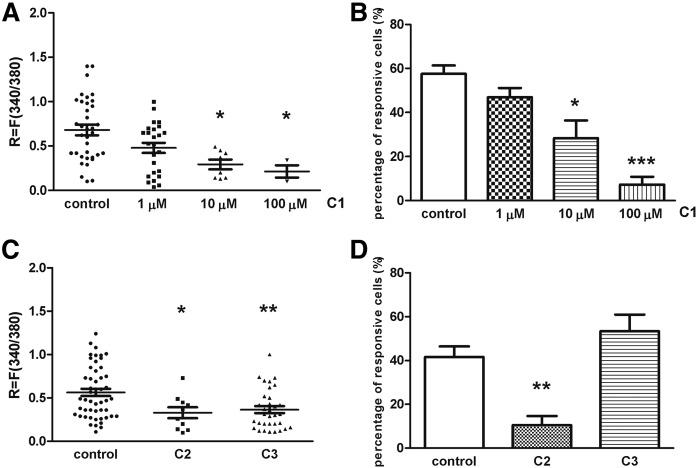
Effect of steroid compounds on TRPV1 receptor activation induced by capsaicin (CAPS) on cultured TG sensory neurons. A: Change in the fluorescence ratio (*R* = F340/F380) is presented after 1, 10, and 100 µM C1 treatment. Dot plot represents mean ± SEM. * *P* < 0.05 (Kruskall-Wallis test with Dunn’s posttest, C1-treated vs. control). n = 32–100 cells per group. B: Percentage of responsive cells to capsaicin is presented after 1, 10, and 100 µM C1 administration. Ca^2+^ responses are presented in percent of total number of examined neurons. * *P* < 0.05; *** *P* < 0.001 (Kruskall-Wallis test with Dunn’s posttest, C1-treated vs. control). n = 32–100 cells per group. C: Change in the fluorescence ratio (*R* = F340/F380) is presented after 100 µM C2 and C3 treatment. Dot plot represents mean ± SEM. * *P* < 0.05; ** *P* < 0.01 (Kruskall-Wallis test with Dunn’s posttest, C2- or C3-treated vs. control). n = 60–96 cells per group. D: Percentage of responsive cells to capsaicin is presented after 100 µM C2 and C3 administration. Ca^2+^ responses are presented in percent of total number of examined neurons. ** *P* < 0.01 (Kruskall-Wallis test with Dunn’s posttest, C2-treated vs. control). n = 60–96 cells per group.

The percent of neurons responding to 330 nM capsaicin was determined in control and steroid compound-treated (C1: 1, 10, and 100 μM; C2: 100 μM; C3: 100 μM) for 45 min in 37°C plates. Application of 330 nM capsaicin for 10 s induced transient Ca^2+^ accumulation in the cytosol of TG neurons as detected by the magnitude of the fluorescence response. Ca^2+^ influx was detected in 57.58 ± 3.78% (36 of 63) of the neurons on control plates of C1. This value did not change in the presence of 1 μM C1, but higher concentrations of steroids caused significant decrease in the proportion of cells responding to capsaicin. These values were 28.33 ± 8.1% (8 of 32) and 7.25 ± 3.4% (3 of 45) after 10 and 100 μM C1, respectively (**Fig. 4B**). Capsaicin-induced fluorescence increment was *R* = 0.68 ± 0.05 on control plates. Incubation with 10 and 100 μM C1 for 45 min at 37°C diminished significantly the capsaicin-evoked fluorescence change, resulting in *R* = 0.29 ± 0.05 and *R* = 0.21 ± 0.06 ratio values, respectively (Fig. 4A). C1 had almost the same inhibitory effect on TRPV1 ion channel activation on TG cells in the presence of serum during the incubation time (capsaicin-induced fluorescence increment was *R* = 0.79 ± 0.06 on control plates, and incubation with 10 μM C1 significantly decreased the capsaicin-evoked fluorescence change resulting in *R* = 0.45 ± 0.1 ratio value; supplemental Fig. S1.). Incubation with C2 (100 μM), but not with C3 (100 μM) caused significant decrease in the proportion of cells responding to capsaicin. These values were 10.48 ± 4.14% (10 of 96) and 53.38 ± 7.59% (33 of 60) after 100 μM C2 and 100 μM C3, respectively (Fig. 4D). Nevertheless, fluorescence increment was diminished both after C2 and C3 administration; *R* values were *R* = 0.32 ± 0.06 and *R* = 0.36 ± 0.04, respectively (Fig. 4C).

### Filipin staining

**Fig. 5.**
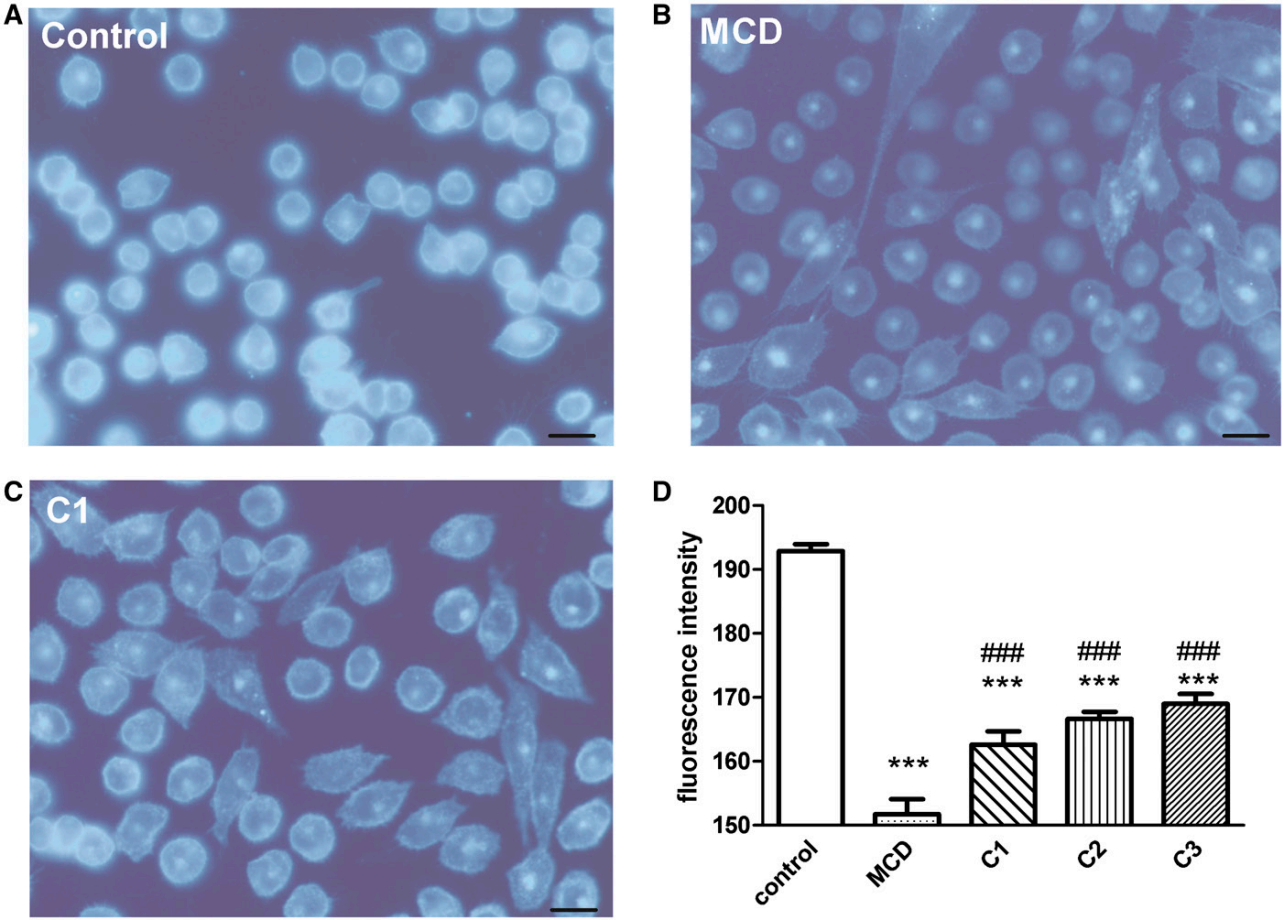
Filipin staining. TRPV1-expressing CHO cell line: control (A), MCD-treated (B), and C1-treated (C). Micrographs have been generated using an Olympus Fluoview-1000 system on an Olympus IX81 microscope stage equipped with an Olympus DP70 digital camera and through an Olympus UPlan FL N, Phase2 objective (40×/0.75). Image size was set at 4080 × 3072 and ISO at 200. Scale bars: 50 µm. D: Quantification of filipin staining by ImageJ software. Fluorescence intensities are presented in percent of control after 10 mM MCD and 100 µM C1, C2, and C3 treatment. *** *P* < 0.001 (one-way ANOVA, Bonferroni’s post hoc test, MCD-, C1-, C2-, or C3-treated vs. control); ^###^ *P* < 0.001 (one-way ANOVA, Bonferroni’s post hoc test, C1-, C2-, or C3-treated vs. MCD-treated). n = at least 120 cells.

CHO cells were stained with the cholesterol-binding compound filipin to visualize at cellular level the effect of steroid compounds on of plasma membrane cholesterol level, as described in macrophages (69). The widely used cholesterol depletor agent MCD (10 mM) served as a positive control beside the steroid treatments. As compared with control cells (**Fig. 5A**), treatment with MCD strongly reduced filipin labeling of the plasma membrane in CHO cells (Fig. 5B). Cholesterol was found in both the plasma membrane and in perinuclear compartments of control cells. Fluorescence intensities were 190 ± 2 and 152 ± 3 in control and MCD-treated plates, respectively (Fig. 5D). Reduction of fluorescence intensity of cholesterol staining was detected in C1-treated (Fig. 5C), C2-treated, and C3-treated CHO cells resulting in 163 ± 3, 167 ± 1, and 169 ± 2 fluorescence intensity values, respectively (Fig. 5D).

### Fluorescence spectroscopy

**Fig. 6.**
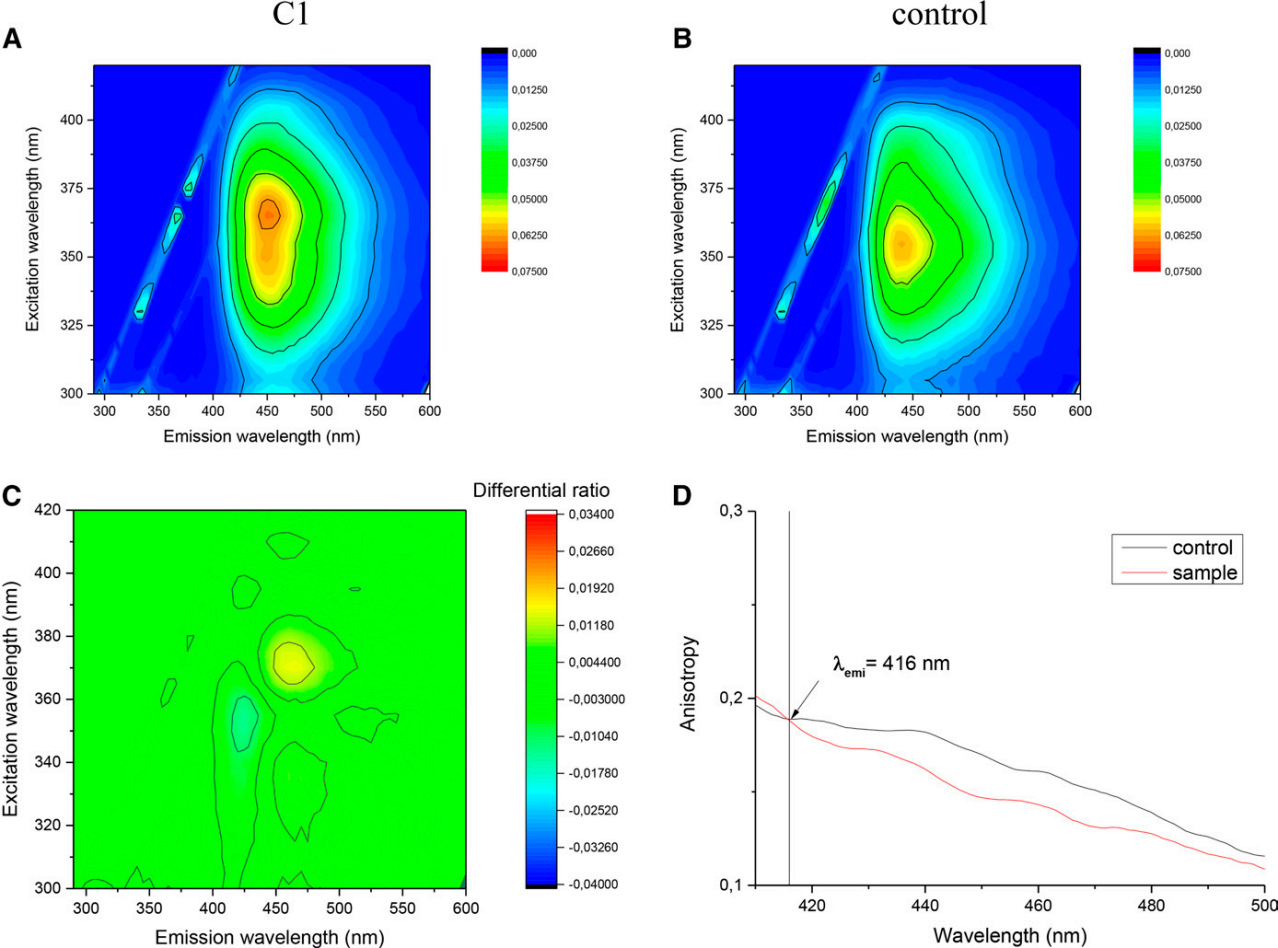
Excitation-emission matrices contour plots of C1-treated sample (A) and its control (B). C: Differential contour plot calculated as A/B. D: Emission anisotropy of C1-treated sample vs. its control.

Laurdan’s fluorescence emission is extremely sensitive for the polarity of the microenvironments (70, 71); thus, laurdan is a suitable fluorophore to distinguish between liquid-ordered (control) and liquid-disordered (treated) membrane phases (74). Shape and spectral position of fluorescence emission and excitation spectra of laurdan depend both on the polarity of its microenvironment and the speed of its dipolar relaxation. In C1-treated sample spectral, broadening and major structural changes can be seen (**Fig. 6A**, B), and the structural change is clearly visible on the difference matrix’ contour plot due to the treatment (Fig. 6C).

To confirm this effect, steady-state emission anisotropy measurements were performed. As Fig. 6D shows, the value of anisotropy (*r*) is significantly lower in the case of disordered phase membrane (sample) than in the case of the ordered phase membrane (control) on the spectral region of laurdan’s emission. This lower anisotropy means that the laurdan’s local motion is less restricted. The lower anisotropy value is the direct evidence of the transition from the ordered toward the disordered membrane phase induced by the sample treatment.

For the C2- and C3-treated samples, the difference between the sample and its control was minimal, which indicates that spectrally no major environmental changes happened in the surroundings of laurdan.

### Effect of steroid compound C1 on TRPA1, TRPM8, and TRPM3 ion channel activation mediated Ca^2+^-influx in cultured TG neurons and AITC-evoked ^45^Ca uptake on CHO cells expressing the cloned TRPA1 receptor

**Fig. 7.**
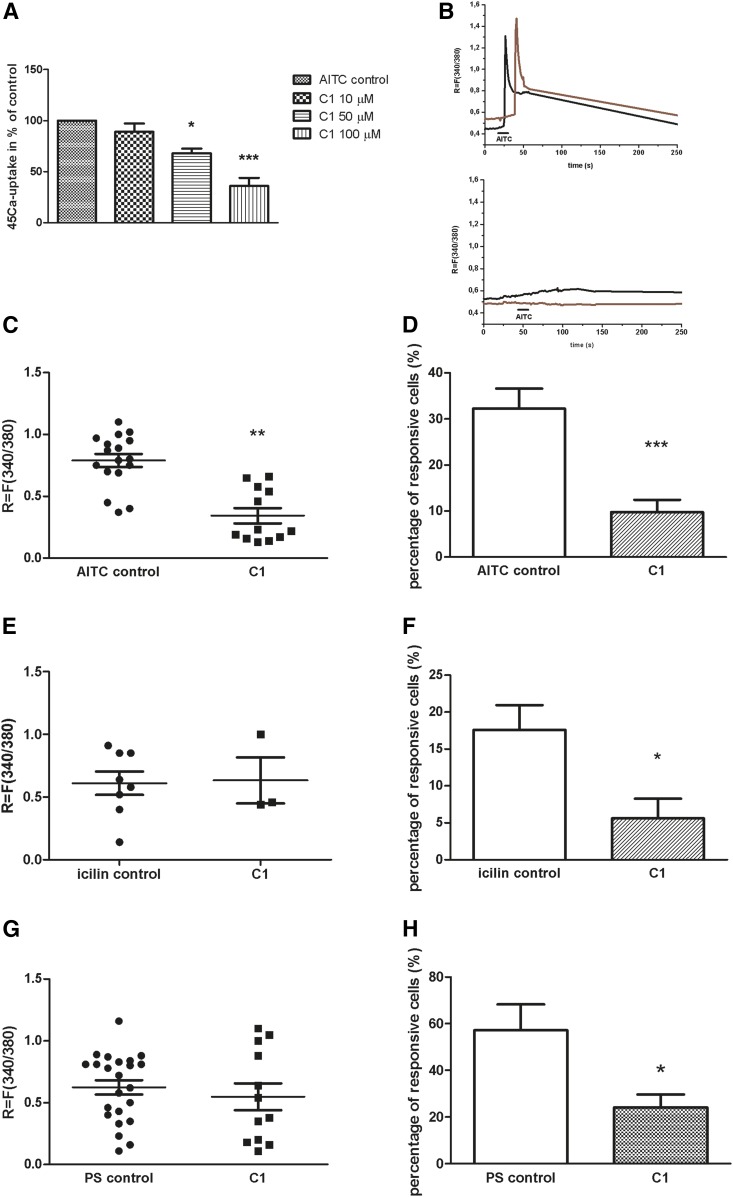
Effect of C1 compound on TRPA1, TRPM8, and TRPM3 receptor activation in cultured TRG neurons and CHO cells expressing the cloned TRPA1 receptor. A: Effect of C1 compound (10, 50, and 100 µM) on AITC (100 µM)-evoked ^45^Ca uptake on CHO cells expressing the cloned TRPA1 receptor. Ca^2+^ accumulations are presented in percent of control (vehicle-treated). Each column represents the mean ± SD of values of three experiments. * *P* < 0.05; *** *P* < 0.001 (one-way ANOVA, Bonferroni’s post hoc test, C1-treated vs. control). B: Increases of *R* = 340/380 fluorescence in fura-2 loaded cultured TG neurons. Upper: Records from 2 AITC-sensitive cells on a control plate. Lower: AITC-induced Ca^2+^ transient in fura-2 loaded cultured TG neurons on a 100 µM C1-treated plate. C: Change in the fluorescence ratio (*R* = F340/F380) in AITC-sensitive cells is presented after 100 µM C1 treatment. Dot plot represents mean ± SEM. ** *P* < 0.01 (Student’s *t*-test for paired comparison, C1-treated vs. control). D: The percentage of responsive cells to AITC is presented after 100 µM C1 administration. Ca^2+^-responses are presented in percent of total number of examined neurons. * *P* < 0.05 (Student’s *t*-test for paired comparison, C1-treated vs. control). n = 51–130 cells per group. E: Change in the fluorescence ratio (*R* = F340/F380) after TRPM8 receptor activation in icilin (1 µM)-sensitive cells is presented after 100 µM C1 treatment. Dot plot represents mean ± SEM. F: Percentage of responsive cells to icilin is presented after 100 µM C1 administration. Ca^2+^-responses are presented in percent of total number of examined neurons. * *P* < 0.05 (Student’s *t*-test for paired comparison, C1-treated vs. control). n = 47–55 cells per group. G: Change in the fluorescence ratio (*R* = F340/F380) after TRPM3 receptor activation in PS (50 µM)-sensitive cells is presented after 100 µM C1 treatment. Dot plot represents mean ± SEM. H: Percentage of responsive cells to PS is presented after 100 µM C1 administration. Ca^2+^ responses are presented in percent of total number of examined neurons. * *P* < 0.05 (Student’s *t*-test for paired comparison, C1-treated vs. control). n = 39–47 cells per group.

In the next series of experiments, C1 steroid compound was used, which exerted the most effective inhibitory action of TRPV1 receptor activation and caused structural changes in plasma membrane as detected by fluorescence spectroscopy. In the presence of 50 µM concentration of C1, intracellular Ca^2+^ influx induced by 100 µM AITC was significantly decreased, measured by the radioactive ^45^Ca uptake method. **Figure 7** shows the percent values of ^45^Ca uptake relative to the vehicle control (100%); C1 in 50 and 100 µM concentrations decreased ^45^Ca uptake to 68 ± 8% and 36 ± 14%, respectively (Fig. 7A).

The percent of neurons responding to 100 μM AITC was determined in control and C1-treated plates with ratiometric technique of intracellular free calcium concentration measurement. The response to AITC had a longer duration and developed after a longer latency (20–30 s). Under control circumstances, Ca^2+^ influx was detected in 32.13 ± 4.3% (17 of 51) of the neurons. Diminution in the proportion of cells responding to AITC was detected after C1 treatment, 9.79 ± 2.68% (12 of 130) of neurons responded with Ca^2+^ influx (Fig. 7D).

AITC-induced fluorescence increment was *R* = 0.79 ± 0.35 on control plates, which was significantly decreased to *R* = 0.35 ± 0.08 (Fig. 7C). Original registrations are shown on Fig. 7B (upper, control; lower, C1-treated).

We investigated whether C1 has any effect on the other two TRP ion channel (TRPM8 and TRPM3) activations, which are involved in pain and thermosensation. The percent of neurons responding with Ca^2+^ influx to the TRPM8 agonist icilin (1 μM) and the TRPM3 agonist PS (50 μM) was determined on control plates. The response to icilin and PS had a long duration after a short latency (5–10 s) in case of icilin and longer latency (20–30 s) in case of PS. The percentages of icilin- and PS-responsive cells were 17.59 ± 3.38% (8 of 47) and 57.35 ± 11.08% (23 of 39) on control plates. A significant decrease in the percent of icilin- and PS-sensitive cells was observed after C1 incubation, resulting in 5.61 ± 2.66% (3 of 55) and 24.09 ± 5.56% (12 of 47) responsive cells (Fig. 7F, H). Peaks of mean fluorescence increments to icilin and PS were measured on control and C1-treated plates. In contrast with the results of the percentage of responsive cells, icilin- and PS-induced Ca^2+^ influx ratio values remained unaltered after C1 treatment (Fig. 7E, G).

### 3.6. Cell viability assays

**Fig. 8.**
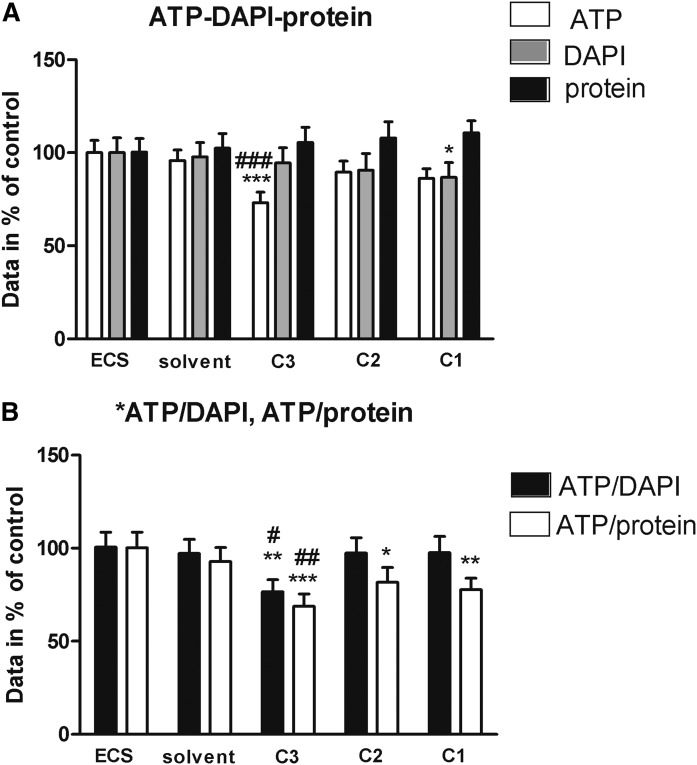
Cell viability assays. ATP, DAPI (cell nuclei), and total protein expression data of cell cultures (A) and the calculated ATP/DAPI and ATP/total protein ratios (B) after C1, C2, and C3 treatment in comparison with untreated (ECS) and solvent-treated controls expressed in percentage. * P < 0.05; ** *P* < 0.01; *** *P* < 0.001 vs. ECS; and ^#^ *P* < 0.05; ^###^ *P* < 0.001 vs. solvent (one-way ANOVA, Bonferroni post hoc test). Slightly decreased cell number was found in case of 100 μM C1.

Regarding ATP concentrations, only 100 μM C3 exposure exerted significant decreasing effect as compared with both ECS and solvent without significant changes in cell number (DAPI data) and total protein contents. Only DAPI staining was diminished minimally, but significantly by C1; C2 did not influence any of these parameters (**Fig. 8A**). The ATP/DAPI and the ATP/protein ratios were more sensitive parameters of viability; only C3 decreased significantly both ratios, but C2 and C1 decreased only the ATP/protein value (Fig. 8B).

## DISCUSSION

We report here that the carboxamido steroid compound C1 significantly inhibits the opening properties of the TRPV1, TRPA1, TRPM8, and TRPM3 cation channels on primary sensory neurons and receptor-expressing cell lines. C1 at a concentration of 10 µM decreased the TRPV1 activation-induced peak of mean fluorescence increment sensory neurons and reduced the ^45^Ca uptake in TRPV1 receptor-expressing cells. C2 having the *N*-(prop-2-ynyl)-carboxamido group in a different position also significantly diminished the TRPV1 ion channel activation, but C3 without the *N*-(prop-2-ynyl)-carboxamido group had a slight inhibitory effect on the gating of the TRPV1 ion channels.

Beyond the position of the carboxamido group in the skeleton of C1 and C2 (C-16 and C-17, respectively), the main difference in the structure of these compounds lies in the unnatural *cis* junction of rings C and D in compound C1, whereas C2 possesses a normal androstane skeleton with a *trans* anellation of the C–D rings. As a result, ring D is forced above the average plane of the steroid in C1, as it was proven in earlier studies by X-ray crystallography (64), whereas C2 bears a quasiplanar structure. In order to study the effect of the molecular structure on the inhibition, a third compound (C3) was also chosen for the investigation. It lacks the *N*-(prop-2-ynyl)-carboxamido group of C1 and C2, but has the same unnatural skeleton as C1. This derivative, a 16-keto-18-nor-13α-steroid (C3), was the starting material during the synthesis of C1. This study revealed that the presence of the carboxamido group, as well as the shape of the steroidal skeleton and the position of the carboxamido group, are important for the development of the inhibitory action on TRP ion channels.

Endogenous steroids have been described as potential modulators of the TRPV1 cation channel. The plant-derived α-spinasterol diminished capsaicin-evoked nociception and edema without affecting body temperature or locomotor activity; thus, it has been described as a novel, effective, and safe TRPV1 inhibitor (75, 76). TRPV1 and TRPM3 ion channels have been linked to neuroactive steroids. Stimulatory and inhibitory effects have been described in case of TRPV1, and stimulatory effects occurred for TRPM3 (27, 63, 77). Stereoselective inhibition of Transient Receptor Potential Canonical 5 (TRPC5) cation channels by steroids has been reported. PS, pregnanolone sulfate, pregnanolone, or dihydrotestosterone diminished the function of TRPC5; progesterone had strong inhibitory effects on TRPC5 (77). DHEA had an inhibitory effect on the TRPV1 channel activation, but its 3α-DHEA stereoisomer potentiated the capsaicin-induced current, suggesting that the interaction of steroids with TRPV1 is stereospecific (63). Analysis of the steroid structure-activity relations for TRP channels is needed, but it is clear that a minimal structural change, including stereo-isomerism, might be enough to change or eliminate the effect (77).

Cell viability decreased moderately, but statistically significantly, only by C3; C1 and C2 did not induce remarkable effect. The observation that the ATP/DAPI and ATP/total protein ratios were more sensitive than the single parameters emphasizes the importance of using at least one reference signal with much less probability to change rapidly (nucleic acid or protein) when determining viability parameters based on ATP assay. A further advantage of the multiparametric approach is that by interpreting all data, one might get information on the potential mode of action of test compounds. It is well known that ATP depletion is reversible down to 20–40% of the initial values, depending on cell type and culture conditions. In this regard, our viability data suggest that C1–C3 treatments did not cause irreversible damage to CHO cells during the relatively short exposure time. It should also be noted that there is always a thorough washing step before the different measurements. Therefore, the detached dead cells are removed first, and the viability data represent only the parameters of the still-attached (postulated to be living) cells. One of the most frequently used viability tests is the measurement of intracellular ATP, but its turnover is extremely rapid and may change inhomogeneous because of the diversity of the living cell population (73).

It can be concluded that our carboxamido steroid compound C1 exerts prominent inhibitory effects on all investigated TRP channels located on primary sensory neurons, such as TRPV1, TRPA1, TRPM3, and TRPM8, and the presence and the position of the carboxamido group are important for this action. We provided the first evidence that our steroid compound C1 is able to deplete cholesterol from the plasma membrane and exerts the same effect in 10 µM concentration as MCD in 1,000 times higher concentration. Therefore, one of the main outcomes of the present experiments is that our new compound C1 is a particularly useful experimental tool to investigate the effect of cholesterol depletion and the subsequent lipid raft disruption on receptor/ion channel functions, and it could replace MCD, which is currently the most widely used compound in this field. The other main message is that the hydrophobic interactions between the TRP channels and lipid rafts can modulate the gating of these ion channels, and targeting this interaction represents a novel and promising drug developmental perspective.

## Supplementary Material

Supplemental Data

## References

[1] Gees M., Owsianik G., Nilius B., and Voets T. 2012 TRP channels. Compr. Physiol. 2: 563–608. 23728980 10.1002/cphy.c110026

[2] Vay L., Gu C., and McNaughton P. A. 2012 The thermo-TRP ion channel family: properties and therapeutic implications. Br. J. Pharmacol. 165: 787–801. 21797839 10.1111/j.1476-5381.2011.01601.x PMC3312478

[3] Szolcsányi J., and Sándor Z. 2012 Multisteric TRPV1 nocisensor: a target for analgesics. Trends Pharmacol. Sci. 33: 646–655. 23068431 10.1016/j.tips.2012.09.002

[4] Welch J. M., Simon S. A., and Reinhart P. H. 2000 The activation mechanism of rat vanilloid receptor 1 by capsaicin involves the pore domain and differs from the activation by either acid or heat. Proc. Natl. Acad. Sci. USA. 97: 13889–13894. 11095706 10.1073/pnas.230146497 PMC17671

[5] Caterina M. J., and Park U. 2006 TRPV1, A polymodal sensor in the nociceptor terminal. *In*: Oh U (Ed) The nociceptive membrane, Academic Press, San Diego, pp 114–150

[6] Myers B. R., Bohlen C. J., and Julius D. 2008 A yeast genetic screen reveals a critical role for the pore helix domain in TRP channel gating. Neuron. 58: 362–373. 18466747 10.1016/j.neuron.2008.04.012 PMC2422846

[7] Szolcsányi J. 2008 Hot target on nociceptors: perspectives, caveats and unique features. Br. J. Pharmacol. 155: 1142–1144. 18997812 10.1038/bjp.2008.374 PMC2607209

[8] Gavva N. R. 2008 Body-temperature maintenance as the predominant function of the vanilloid receptor TRPV1. Trends Pharmacol. Sci. 29: 550–557. 18805596 10.1016/j.tips.2008.08.003

[9] Raisinghani M., Pabbidi R. M., and Premkumar L. S. 2005 Activation of transient receptor potential vanilloid 1 (TRPV1) by resiniferatoxin. J. Physiol. 567: 771–786. 16037081 10.1113/jphysiol.2005.087874 PMC1474234

[10] Smart D., Gunthorpe M. J., Jerman J. C., Nasir S., Gray J., Muir A. I., Chambers J. K., Randall A. D., and Davis J. B. 2000 The endogenous lipid anandamide is a full agonist at the human vanilloid receptor (hVR1). Br. J. Pharmacol. 129: 227–230. 10694225 10.1038/sj.bjp.0703050 PMC1571834

[11] Hwang S. W., Cho H., Kwak J., Lee S. Y., Kang C. J., Jung J., Cho S., Min K. H., Suh Y. G., Kim D., 2000 Direct activation of capsaicin receptors by products of lipoxygenases: en dogenous capsaicin-like substances. Proc. Natl. Acad. Sci. USA. 97: 6155–6160. 10823958 10.1073/pnas.97.11.6155 PMC18574

[12] Bianchi B. R., Lee C. H., Jarvis M. F., El Kouhen R., Moreland R. B., Faltynek C. R., and Puttfarcken P. S. 2006 Modulation of human TRPV1 receptor activity by extracellular protons and host cell expression system. Eur. J. Pharmacol. 537: 20–30. 16630609 10.1016/j.ejphar.2006.03.003

[13] Cao E., Cordero-Morales J. F., Liu B., Qin F., and Julius D.. TRPV1 channels are intrinsically heat sensitive and negatively regulated by phosphoinositide lipids 2013 Neuron. 77: 667–679. 23439120 10.1016/j.neuron.2012.12.016 PMC3583019

[14] Story G. M., Peier A. M., Reeve A. J., Eid S. R., Mosbacher J., Hricik T. R., Earley T. J., Hergarden A. C., Andersson D. A., Hwang S. W., 2003 ANKTM1, a TRP-like channel expressed in nociceptive neurons, is activated by cold temperatures. Cell. 112: 819–829. 12654248 10.1016/s0092-8674(03)00158-2

[15] Corey D. P., García-Añoveros J., Holt J. R., Kwan K. Y., Lin S. Y., Vollrath M. A., Amalfitano A., Cheung E. L., Derfler B. H., Duggan A., 2004 TRPA1 is a candidate for the mechanosensitive transduction channel of vertebrate hair cells. Nature. 432: 723–730. 15483558 10.1038/nature03066

[16] Vilceanu D., and Stucky C. L. 2010 TRPA1 mediates mechanical currents in the plasma membrane of mouse sensory neurons. PLoS One. 5: e12177. 20808441 10.1371/journal.pone.0012177 PMC2922334

[17] Bandell M., Story G. M., Hwang S. W., Viswanath V., Eid S. R., Petrus M. J., Earley T. J., and Patapoutian A. 2004 Noxious cold ion channel TRPA1 is activated by pungent compounds and bradykinin. Neuron. 41: 849–857. 15046718 10.1016/s0896-6273(04)00150-3

[18] Jordt S. E., Bautista D. M., Chuang H. H., McKemy D. D., Zygmunt P. M., Högestätt E. D., Meng I. D., and Julius D. 2004 Mustard oils and cannabinoids excite sensory nerve fibres through the TRP channel ANKTM1. Nature. 427: 260–265. 14712238 10.1038/nature02282

[19] McNamara C. R., Mandel-Brehm J., Bautista D. M., Siemens J., Deranian K. L., Zhao M., Hayward N. J., Chong J. A., Julius D., Moran M. M., 2007 TRPA1 mediates formalin-induced pain. Proc. Natl. Acad. Sci. USA. 104: 13525–13530. 17686976 10.1073/pnas.0705924104 PMC1941642

[20] Macpherson L. J., Geierstanger B. H., Viswanath V., Bandell M., Eid S. R., Hwang S., and Patapoutian A. 2005 The pungency of garlic: activation of TRPA1 and TRPV1 in response to allicin. Curr. Biol. 15: 929–934. 15916949 10.1016/j.cub.2005.04.018

[21] Macpherson L. J., Dubin A. E., Evans M. J., Marr F., Schultz P. G., Cravatt B. F., and Patapoutian A. 2007 Noxious compounds activate TRPA1 ion channels through covalent modification of cysteines. Nature. 445: 541–545. 17237762 10.1038/nature05544

[22] Trevisani M., Siemens J., Materazzi S., Bautista D. M., Nassini R., Campi B., Imamachi N., Andrè E., Patacchini R., Cottrell G. S., 2007 4-Hydroxynonenal, an endogenous aldehyde, causes pain and neurogenic inflammation through activation of the irritant receptor TRPA1. Proc. Natl. Acad. Sci. USA. 104: 13519–13524. 17684094 10.1073/pnas.0705923104 PMC1948902

[23] Bautista D. M., Pellegrino M., and Tsunozaki M. 2013 TRPA1: A gatekeeper for inflammation. Annu. Rev. Physiol. 75: 181–200. 23020579 10.1146/annurev-physiol-030212-183811 PMC4041114

[24] Grimm C., Kraft R., Sauerbruch S., Schultz G., and Harteneck C. 2003 Molecular and functional characterization of the melastatin-related cation channel TRPM3. J. Biol. Chem. 278: 21493–21501. 12672799 10.1074/jbc.M300945200

[25] Lee N., Chen J., Sun L., Wu S., Gray K. R., Rich A., Huang M., Lin J. H., Feder J. N., Janovitz E. B., 2003 Expression and characterization of human transient receptor potential melastatin 3 (hTRPM3). J. Biol. Chem. 278: 20890–20897. 12672827 10.1074/jbc.M211232200

[26] Oberwinkler J., and Philipp S. E. 2014 TRPM3. Handb. Exp. Pharmacol. 222: 427–459. 24756716 10.1007/978-3-642-54215-2_17

[27] Wagner T. F., Loch S., Lambert S., Straub I., Mannebach S., Mathar I., Düfer M., Lis A., Flockerzi V., Philipp S. E., 2008 Transient receptor potential M3 channels are ionotropic steroid receptors in pancreatic beta cells. Nat. Cell Biol. 10: 1421–1430. 18978782 10.1038/ncb1801

[28] Drews A., Mohr F., Rizun O., Wagner T. F., Dembla S., Rudolph S., Lambert S., Konrad M., Philipp S. E., Behrendt M., 2014 Structural requirements of steroidal agonists of transient receptor potential melastatin 3 (TRPM3) cation channels. Br. J. Pharmacol. 171: 1019–1032. 24251620 10.1111/bph.12521 PMC3925040

[29] Vriens J., Owsianik G., Hofmann T., Philipp S. E., Stab J., Chen X., Benoit M., Xue F., Janssens A., Kerselaers S., 2011 TRPM3 is a nociceptor channel involved in the detection of noxious heat. Neuron. 70: 482–494. 21555074 10.1016/j.neuron.2011.02.051

[30] Majeed Y., Agarwal A. K., Naylor J., Seymour V. A., Jiang S., Muraki K., Fishwick C. W., and Beech D. J. 2010 Cis-isomerism and other chemical requirements of steroidal agonists and partial agonists acting at TRPM3 channels. Br. J. Pharmacol. 161: 430–441. 20735426 10.1111/j.1476-5381.2010.00892.x PMC2989593

[31] Grimm C., Kraft R., Schultz G., and Harteneck C. 2005 Activation of the melastatin-related cation channel TRPM3 by D-erythro-sphingosine. [corrected] Mol. Pharmacol. 67: 798–805. 15550678 10.1124/mol.104.006734

[32] Vriens J., Held K., Janssens A., Tóth B. I., Kerselaers S., Nilius B., Vennekens R., and Voets T. 2014 Opening of an alternative ion permeation pathway in a nociceptor TRP channel. Nat. Chem. Biol. 10: 188–195. 24390427 10.1038/nchembio.1428

[33] Reid G., and Flonta M. L. 2002 Ion channels activated by cold and menthol in cultured rat dorsal root ganglion neurones. Neurosci. Lett. 324: 164–168. 11988352 10.1016/s0304-3940(02)00181-7

[34] McKemy D. D., Neuhausser W. M., and Julius D. 2002 Identification of a cold receptor reveals a general role for TRP channels in thermosensation. Nature. 416: 52–58. 11882888 10.1038/nature719

[35] Peier A. M., Moqrich A., Hergarden A. C., Reeve A. J., Andersson D. A., Story G. M., Earley T. J., Dragoni I., McIntyre P., Bevan S., 2002 A TRP Channel that Senses Cold Stimuli and Menthol. Cell. 108: 705–715. 11893340 10.1016/s0092-8674(02)00652-9

[36] Bautista D. M., Siemens J., Glazer J. M., Tsuruda P. R., Basbaum A. I., Stucky C. L., Jordt S. E., and Julius D. 2007 The menthol receptor TRPM8 is the principal detector of environmental cold. Nature. 448: 204–208. 17538622 10.1038/nature05910

[37] Bandell M., Dubin A. E., Petrus M. J., Orth A., Mathur J., Hwang S. W., and Patapoutian A. 2006 High-throughput random mutagenesis screen reveals TRPM8 residues specifically required for activation by menthol. Nat. Neurosci. 9: 493–500. 16520735 10.1038/nn1665

[38] Chuang H. H., Neuhausser W. M., and Julius D. 2004 The super-cooling agent icilin reveals a mechanism of coincidence detection by a temperature-sensitive TRP channel. Neuron. 43: 859–869. 15363396 10.1016/j.neuron.2004.08.038

[39] Akopian A. N., Ruparel N. B., Patwardhan A., and Hargreaves K. M. 2008 Cannabinoids desensitize capsaicin and mustard oil responses in sensory neurons via TRPA1 activation. J. Neurosci. 28: 1064–1075. 18234885 10.1523/JNEUROSCI.1565-06.2008 PMC6671418

[40] Salas M. M., Hargreaves K. M., and Akopian A. N. 2009 TRPA1-mediated responses in trigeminal sensory neurons: interaction between TRPA1 and TRPV1. Eur. J. Neurosci. 29: 1568–1578. 19419422 10.1111/j.1460-9568.2009.06702.x PMC2765106

[41] Helyes Z., Németh J., Thán M., Bölcskei K., Pintér E., and Szolcsányi J. 2003 Inhibitory effect of anandamide on resiniferatoxin-induced sensory neuropeptide release in vivo and neuropathic hyperalgesia in the rat. Life Sci. 73: 2345–2353. 12941436 10.1016/s0024-3205(03)00651-9

[42] Helyes Z., Pintér E., Sándor K., Elekes K., Bánvölgyi A., Keszthelyi D., Szoke E., Tóth D. M., Sándor Z., Kereskai L., 2009 Impaired defense mechanism against inflammation, hyperalgesia, and airway hyperreactivity in somatostatin 4 receptor gene-deleted mice. Proc. Natl. Acad. Sci. USA. 106: 13088–13093. 19622729 10.1073/pnas.0900681106 PMC2722291

[43] Szolcsányi J. 2004 Forty years in capsaicin research for sensory pharmacology and physiology. Neuropeptides. 38: 377–384. 15567473 10.1016/j.npep.2004.07.005

[44] Moran M. M., McAlexander M. A., Bíró T., and Szallasi A. 2011 Transient receptor potential channels as therapeutic targets. Nat. Rev. Drug Discov. 10: 601–620. 21804597 10.1038/nrd3456

[45] Nilius B., and Szallasi A. 2014 Transient receptor potential channels as drug targets: from the science of basic research to the art of medicine. Pharmacol. Rev. 66: 676–814. 24951385 10.1124/pr.113.008268

[46] Kaneko Y., and Szallasi A. 2014 Transient receptor potential (TRP) channels: a clinical perspective. Br. J. Pharmacol. 171: 2474–2507. 24102319 10.1111/bph.12414 PMC4008995

[47] Helyes Z., Pintér E., Németh J., and Szolcsányi J. 2003 Pharm­acological targets for the inhibition of neurogenic inflammation. Curr. Med. Chem. 2: 191–218.

[48] Lee Y., Hong S., Cui M., Sharma P. K., Lee J., and Choi S. 2015 Transient receptor potential vanilloid type 1 antagonists: a patent review (2011–2014). Expert Opin. Ther. Pat. 25: 291–318. 25666693 10.1517/13543776.2015.1008449

[49] Corbin J., Wang H. H., and Blanton M. P. 1998 Identifying the cholesterol binding domain in the nicotinic acetylcholine receptor with [125I]azido-cholesterol. Biochim. Biophys. Acta. 1414: 65–74. 9804895 10.1016/s0005-2736(98)00153-9

[50] Simons K., and Toomre D. 2000 Lipid rafts and signal transduction. Nat. Rev. Mol. Cell Biol. 1: 31–39. 11413487 10.1038/35036052

[51] Mishra S., and Joshi P. G. 2007 Lipid raft heterogeneity: an enigma. J. Neurochem. 103: 135–142. 17986148 10.1111/j.1471-4159.2007.04720.x

[52] Sjögren B., and Svenningsson P. 2007 Depletion of the lipid raft constituents, sphingomyelin and ganglioside, decreases serotonin binding at human 5–HT7(a) receptors in HeLa cells. Acta Physiol. (Oxf.). 190: 47–53. 17428232 10.1111/j.1365-201X.2007.01687.x

[53] Colón-Sáez J. O., and Yakel J. L. 2011 The α7 nicotinic acetylcholine receptor function in hippocampal neurons is regulated by the lipid composition of the plasma membrane. J. Physiol. 589: 3163–3174. 21540349 10.1113/jphysiol.2011.209494 PMC3145932

[54] Bergdahl A., Gomez M. F., Dreja K., Xu S. Z., Adner M., Beech D. J., Broman J., Hellstrand P., and Sward K. 2003 Cholesterol depletion impairs vascular reactivity to endothelin-1 by reducing store-operated Ca2+ entry dependent on TRPC1. Circ. Res. 93: 839–847. 14551243 10.1161/01.RES.0000100367.45446.A3

[55] Graziani A., Rosker C., Kohlwein S. D., Zhu M. X., Romanin C., Sattler W., Groschner K., and Poteser M. 2006 Cellular cholesterol controls TRPC3 function: evidence from a novel dominant-negative knockdown strategy. Biochem. J. 396: 147–155. 16448384 10.1042/BJ20051246 PMC1449990

[56] Lockwich T. P., Liu X., Singh B. B., Jadlowiec J., Weiland S., and Ambudkar I. S. 2000 Assembly of Trp1 in a signaling complex associated with caveolin-scaffolding lipid raft domains. J. Biol. Chem. 275: 11934–11942. 10766822 10.1074/jbc.275.16.11934

[57] Liu B., Hui K., and Qin F. 2003 Thermodynamics of heat activation of single capsaicin ion channels VR1. Biophys. J. 85: 2988–3006. 14581201 10.1016/S0006-3495(03)74719-5 PMC1303577

[58] Liu M., Huang W., Wu D., and Priestley J. V. 2006 TRPV1, but not P2X3, requires cholesterol for its function and membrane expression in rat nociceptors. Eur. J. Neurosci. 24: 1–6. 16800863 10.1111/j.1460-9568.2006.04889.x

[59] Bari M., Battista N., Fezza F., Finazzi-Agrò A., and Maccarrone M. 2005 Lipid rafts control signaling of type-1 cannabinoid receptors in neuronal cells: implications for anandamide-induced apoptosis. J. Biol. Chem. 280: 12212–12220. 15657045 10.1074/jbc.M411642200

[60] Morenilla-Palao C., Pertusa M., Meseguer V., Cabedo H., and Viana F. 2009 Lipid raft segregation modulates TRPM8 channel activity. J. Biol. Chem. 284: 9215–9224. 19176480 10.1074/jbc.M807228200 PMC2666574

[61] Szoke E., Börzsei R., Tóth D. M., Lengl O., Helyes Z., Sándor Z., and Szolcsányi J. 2010 Effect of lipid raft disruption on TRPV1 receptor activation of trigeminal sensory neurons and transfected cell line. Eur. J. Pharmacol. 628: 67–74. 19958765 10.1016/j.ejphar.2009.11.052

[62] Sághy É., Szőke É., Payrits M., Helyes Z., Börzsei R., Erostyák J., Jánosi T. Z., Sétáló G. Jr., and Szolcsányi J. 2015 Evidence for the role of lipid rafts and sphingomyelin in Ca2+-gating of Transient Receptor Potential channels in trigeminal sensory neurons and peripheral nerve terminals. Pharmacol. Res. 100: 101–116. 26238178 10.1016/j.phrs.2015.07.028

[63] Chen S. C., Chang T. J., and Wu F. S. 2004 Competitive inhibition of the capsaicin receptor-mediated current by dehydroepiandrosterone in rat dorsal root ganglion neurons. J. Pharmacol. Exp. Ther. 311: 529–536. 15201344 10.1124/jpet.104.069096

[64] Szánti-Pintér E., Wouters J., Gömöry Á., Sághy É., Szőke É., Helyes Z., Kollár L., and Skoda-Földes R. 2015 Synthesis of novel 13α-18-norandrostane-ferrocene conjugates via homogeneous catalytic methods and their investigation on TRPV1 receptor activation. Steroids. 104: 284–293. 26519768 10.1016/j.steroids.2015.10.016

[65] Szőke E., Balla Z., Csernoch L., Czéh G., and Szolcsányi J. 2000 Interacting effects of capsaicin and anandamide on intracellular calcium in sensory neurons. Neuroreport. 11: 1949–1952. 10884049 10.1097/00001756-200006260-00028

[66] Sándor Z., Varga A., Horváth P., Nagy B., and Szolcsányi J. 2005 Construction of a stable cell line uniformly expressing the rat TRPV1 receptor. Cell. Mol. Biol. Lett. 10: 499–514. 16217559

[67] Horváth A., Szájli Á., Kiss R., Kóti J., Mahó S., and Skoda-Földes R. 2011 Ionic liquid promoted Wagner-Meerwein rearrangement of 16α,17α-epoxy androstanes and estranes. J. Org. Chem. 76: 6048–6056. 21668005 10.1021/jo2006285

[68] Szánti-Pintér E., Balogh J., Csók Z., Kollár L., Gömöry Á., and Skoda-Földes R. 2011 Synthesis of steroid-ferrocene conjugates of steroidal 17-carboxamides via a palladium-catalyzed aminocarbonylation – copper-catalyzed azide-alkyne cycloaddition reaction sequence. Steroids. 76: 1377–1382. 21787798 10.1016/j.steroids.2011.07.006

[69] Tabas I., Zha X., Beatini N., Myers J. N., and Maxfield F. R. 1994 The actin cytoskeleton is important for the stimulation of cholesterol esterification by atherogenic lipoproteins in macrophages. J. Biol. Chem. 269: 22547–22556. 8077203

[70] Harris F. M., Best K. B., and Bell J. D. 2002 Use of laurdan fluorescence intensity and polarization to distinguish between changes in membrane fluidity and phospholipid order. Biochim. Biophys. Acta. 1565: 123–128. 12225860 10.1016/s0005-2736(02)00514-x

[71] Gaus K., Gratton E., Kable E. P., Jones A. S., Gelissen I., Kritharides L., and Jessup W. 2003 Visualizing lipid structure and raft domains in living cells with two-photon microscopy. Proc. Natl. Acad. Sci. USA. 100: 15554–15559. 14673117 10.1073/pnas.2534386100 PMC307606

[72] Koszegi T., Petrik J., Vladimir-Kneževič S., and Nagy S. 2007 Co determination of ATP and proteins in Triton X 100 non-ionic detergent-opened monolayer cultured cells. Luminescence. 22: 415–419. 17516425 10.1002/bio.979

[73] Sali N., Nagy S., Poór M., and Kőszegi T. 2016 Multiparametric luminescent cell viability assay in toxicology models: A critical evaluation. J. Pharmacol. Toxicol. Methods. 79: 45–54. 26777595 10.1016/j.vascn.2016.01.004

[74] Golfetto O., Hinde E., and Gratton E. 2013 Laurdan fluorescence lifetime discriminates cholesterol content from changes in fluidity in living cell membranes. Biophys. J. 104: 1238–1247. 23528083 10.1016/j.bpj.2012.12.057 PMC3602759

[75] Trevisan G., Rossato M. F., Walker C. I., Klafke J. Z., Rosa F., Oliveira S. M., Tonello R., Guerra G. P., Boligon A. A., Zanon R. B., 2012 Identification of the plant steroid α-spinasterol as a novel transient receptor potential vanilloid 1 antagonist with antinociceptive properties. J. Pharmacol. Exp. Ther. 343: 258–269. 22837009 10.1124/jpet.112.195909

[76] Socała K., Nieoczym D., Pieróg M., and Wlaź P. 2015 α-Spinasterol, a TRPV1 receptor antagonist, elevates the seizure threshold in three acute seizure tests in mice. J. Neural Transm. (Vienna). 122: 1239–1247 (Vienna). 25764210 10.1007/s00702-015-1391-7 PMC4540766

[77] Majeed Y., Amer M. S., Agarwal A. K., McKeown L., Porter K. E., O’Regan D. J., Naylor J., Fishwick C. W., Muraki K., and Beech D. J. 2011 Stereo-selective inhibition of transient receptor potential TRPC5 cation channels by neuroactive steroids. Br. J. Pharmacol. 162: 1509–1520. 21108630 10.1111/j.1476-5381.2010.01136.x PMC3057289

