# Supplementary material for: Carboxamido steroids inhibit the opening properties of transient receptor potential ion channels by lipid raft modulation
Source: J Lipid Res. 2018 Aug 9;59(10):1851–63. doi: 10.1194/jlr.M084723 (PMC6168298; doi:10.1194/jlr.M084723)
Supplement: Supplemental Data [file supp_59_10_1851__index.html]

Carboxamido steroids inhibit the opening properties of Transient Receptor Potential ion channels by lipid raft modulation — Carboxamido steroids inhibit the opening properties of transient receptor potential ion channels by lipid raft modulation — Supplemental Data 

# Carboxamido steroids inhibit the opening properties of transient receptor potential ion channels by lipid raft modulation

## Supplemental Data

- Supplement Figure 1 (.pdf, 236 KB) - Effect of C1 on TRPV1 receptor activation induced by capsaicin on cultured TG sensory neurons in the presence of serum during the incubation time
