# Supplementary material for: Carboxamido steroids inhibit the opening properties of transient receptor potential ion channels by lipid raft modulation
Source: J Lipid Res. 2018 Aug 9;59(10):1851–63. doi: 10.1194/jlr.M084723 (PMC6168298; doi:10.1194/jlr.M084723)
Supplement: Supplemental Data [file 10.1194_M084723_jlr.M084723-1.pdf]

Carboxamido steroids inhibit the opening properties of Transient Receptor Potential ion channels by lipid raft modulation

Éva Sághy[1,2,4], Maja Payrits[1,2], Tünde Bíró-Sütő[1,2], Rita Skoda-Földes[5], Eszter Szánti-Pintér[5], János Erostyák[2,6], Géza Makkai[2,6], György Sétáló Jr[2,7], László Kollár[8], Tamás Kőszegi[2,9], Rita Csepregi[2,9], János Szolcsányi[1,2], Zsuzsanna Helyes[1,2,3*], Éva Szőke[1,2,3*]

[1]*Department of Pharmacology and Pharmacotherapy, Medical School, University of Pécs, Pécs; Hungary,* [2]*János Szentágothai Research Center & Centre for Neuroscience, University of Pécs; Hungary,* [3] National Brain Research Program-2 *Chronic Pain Research Group, Pécs; Hungary,* [4]*Semmelweis University, Department of Pharmacology and Pharmacotherapy,*[5]*University of Pannonia, Institute of Chemistry, Department of Organic Chemistry, Veszprém, Hungary* [6]*Department of Experimental Physics, University of Pécs, Pécs; Hungary,* [7]*Department of Medical Biology, Medical School, University of Pécs, Pécs; Hungary,* [8]*Department of Inorganic Chemistry and MTA-PTE Research Group for Selective Chemical Syntheses, University of Pécs, Pécs; Hungary,* [9]*Department of Laboratory Medicine, Medical School, University of Pécs, Pécs; Hungary*

Running title: **Carboxamido steroids inhibit TRP channels by lipid raft modulation**

*Éva Szőke and Zsuzsanna Helyes contributed equally in this study

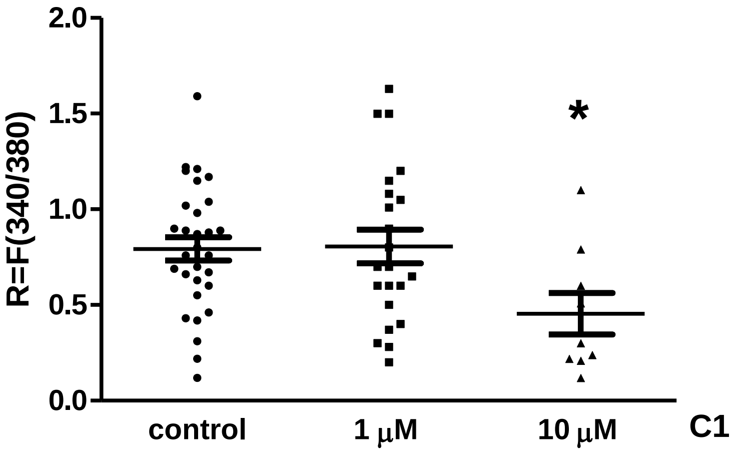

Supplement Figure S1: Effect of C1 on TRPV1 receptor activation induced by capsaicin on cultured TG sensory neurons in the presence of serum during the incubation time: Change in the fluorescence ratio (R = F340/F380) is presented after 1 and 10 μM C1 treatment. Dot plot represents mean±SEM **P*<0.05 (Kruskall-Wallis test with Dunn's post test, C1-treated vs. control). N=50-90 cells per group.
